# On the Atomic Weight of Gallium

**DOI:** 10.6028/jres.081A.002

**Published:** 1977-02-01

**Authors:** George Marinenko

**Affiliations:** Institute for Materials Research, National Bureau of Standards, Washington, D. C. 20234

**Keywords:** Atomic weight, coulometry, electrochemical equivalent, gallium arsenide, gallium atomic weight, stoichiometry

## Abstract

Accurate measurement of stoichiometry of GaAs provides new data which are used for calculation of the atomic weight of Ga. Using the IUPAC accepted value for the atomic weight of As (74.9216), the atomic weight of Ga based on this work is 69.737 ± 0.006. The mean of two independent chemical values for the atomic weight of Ga, one obtained by Lundell and Hoffman and the other in this work, is 69.735.

## 1. Introduction

The presently accepted value of the atomic weight of gallium is 69.72 [1].^1^ This value is based primarily on the work of Richards and Craig [2, 3] involving preparation of gallium trichloride with subsequent determination of GaCl_3_/3Ag ratio, conversion of gallium metal to gallium oxide via several routes by Lundell and Hoffman [4] and mass spectrometric determination of ^69^Ga/^71^Ga ratio by Inghram, et al. [5]. It is interesting that the “rounded value” of the atomic weight of Ga as reported by Lundell and Hoffman [4] is 69.74 which differs significantly from 69.716, the value obtained on the basis GaCl_3_/3Ag ratio and from the mass spectrometric value of 69.72.

The establishment of atomic weights has never been easy in view of high demands on accuracy and the need for many independent methods of determining these “constants.” In some cases the values based on the mean of many measurements were found to agree quite well with the data obtained by new methods and instrumentation used for such purposes. Such is the case with potassium, for which the newly determined atomic weight by means of absolute mass spectrometric isotopic abundance measurement [6] is in excellent agreement with the atomic weight of potassium based on chemical intercomparison data [7].

DeLaeter recently reported a mass-spectrometrically measured ^71^Ga/^69^Ga ratio as 0.6559 [8]. The mass spectrometer used in this work produces isotope fractionation, resulting in the apparent lighter isotope (^69^Ga) enrichment. The magnitude of this effect, as measured by DeLaeter from ^87^Rb/^85^Rb for the National Bureau of Standards rubidium reference material SRM 984, is 0.625 percent per mass unit difference. Thus applying an overall fractionation correction of 1.25 percent to gallium isotope ratio the corrected value was reported as 0.6641. This ratio, in turn, when combined with isotopic mass data [9], yields 69.724 as the atomic weight of gallium.

Coulometry, by no means a new method, has been used for accurate determination of atomic weights as recently as 1975 [10, 11], and as a method of preference for the assay of the separated isotope solutions for absolute calibration of mass spectrometers, used in atomic weight determinations [12, 13, 14, 15]. In this paper data are reported which were obtained as part of a different experiment, namely the evaluation of the stoichiometry of a compound, through the determination of major constituents. These data on coulometric determinations of stoichiometry of GaAs shed some additional light on the atomic weight of Ga.

## 2. Material

As part of a chemical characterization exchange program under the auspices of OECD (Organization for Economic Cooperation and Development), characterization of high purity, specially prepared single crystal gallium arsenide was one of the problems of interest [16]. The material had been prepared by a state-of-the-art procedure to produce single crystals of high purity and accurate stoichiometry. The determination of impurities in this material by other methods indicates that it is of extremely high purity. For the purpose at hand even 99.995 percent purity would have been acceptable, but of course the material used is of higher purity. For example, mass spectrometric determination of impurities indicates that by difference gallium and arsenic comprise 99.9993 weight percent of this material, while neutron activation determination of impurities indicates that gallium and arsenic constitute 99.9994 weight percent [16].

Four different samples of single crystal GaAs were investigated. Samples, weighing on the average 320 mg, were removed from their plastic packaging, placed on a porcelain plate and divided approximately in half by applying moderate pressure with a steel knife along the cleavage plane of the crystal. Subsequently each piece was etched in a platinum crucible using ACS reagent-grade HF, washed in distilled water and vacuum dried in a desiccator as specified in the OECD instructions under deoxygenation procedure. Each piece was placed in a platinum boat, weighed and delivered carefully into a previously washed and dried 125 ml Erlenmeyer flask. Five milliliters of “Suprapure” nitric acid were delivered into the flask in a hood, the flask was inclined so that it was virtually horizontal, to prevent the loss of spray, and left in that position until dissolution of the sample was complete.

## 3. Method

### 3.1 General Description

The general classification of the method is constant current coulometric titration with amperometric end-point determination. Since this study involved the determination of GaAs stoichiometry obviously two methods were used: one for Ga and one for As. Gallium was determined via an EDTA (ethylenediamine tetraacetic acid) complexation reaction and back titration of excess EDTA with zinc. In general, the analytical procedure of Reilley et al. was adopted [17]. Reilley et al. have shown that gallium can be determined volumetrically with precision and accuracy better than 0.05 percent. The coulometric procedure, enabling even more accurate quantitation, was developed in this laboratory. With the exception of the reduction procedure arsenic determination was based on our earlier work [18].

Reilley’s procedure for the determination of gallium requires standard EDTA and standard zinc solutions. Excess EDTA was added to complex the trivalent gallium (in acetate buffer solution), heated, and the excess EDTA was back titrated with standard zinc solution. A small amount of mercuric EDTA complex was added to the solution to act as an indicator ion, using a mercury indicator electrode.

In our method, EDTA was handled as a solid. It was standardized coulometrically with electrogenerated Zn^++^. The electrochemical behavior of Zn (Hg) was reported earlier [8, 9]. The end-point of this titration was determined amperometrically using a gold amalgam polarizable electrode, a standard calomel reference electrode and an applied potential of 1.20 V versus SCE (saturated colomel electrode). This potential corresponds to the diffusion plateau of the Zn^++^ reduction wave.

### 3.2 Coulometric Standardization of EDTA

In order to proceed with determinations of gallium via the EDTA titration, it is necessary to know the “complexometric titer” of EDTA. It was, therefore, decided to determine the electrochemical equivalent of a small lot of Na_2_EDTA·2H_2_O for this purpose. The material (about 500 g) was stored in a 52 percent relative humidity atmosphere (over saturated Mg(NO_3_)_2_ solution). A two gram sample of the material was used for each determination. After weighing EDTA samples by substitution they were delivered into 125 ml Erlenmeyer flasks.

After deaeration of the electrolyte (0.5 M NaCl + 0.5 M sodium acetate) a small amount of EDTA (e.g. 30 *μ*eq) was added to the anode compartment and the electrolyte was pretitrated with electrogenerated Zn^++^ to an amperometric end point. The electrolyte was then permitted to flow into the intermediate compartments of the cell and also drawn into the Erlenmeyer flask (equipped with siphon arrangement) containing the weighed EDTA sample. The sample was dissolved and forced into the cell. Subsequently, sufficient Zn^++^ was generated at 100 mA to react with about 99.8 percent of the calculated amount of EDTA in the sample. The passage of current through the cell was then stopped. The Erlenmeyer flask, which originally contained the sample, was rinsed by repeated filling and emptying of the electrolyte from the cell, and the intermediate cell compartments were rinsed by appropriate manipulation of the vacuum and nitrogen lines, leaving only a small amount of electrolyte on the bottom of these compartments to permit the passage of low current. Then the titration was resumed. At this stage, just as in pretitration, the titration was carried out at 6.43 mA current. An argon stream was bubbled through the solution and the indicator current was measured after the passage of each increment of charge (1 *μ*eq of Zn^++^). After the completion of the data acquisition in the end-point region and beyond it, the two linear portions of the indicator current (residual current and the limiting current due to the reduction wave of Zn^++^) were extrapolated graphically to the point of intersection. Since the indicator current exhibits a significant curvature in the end-point region and the slopes of the indicator current lines are not very reproducible, such an extrapolation could cause some significant errors. Consequentlv, the point of maximum change in the slope of the indicator current function was approximated by bisection of the angle between the residual current and Zn^++^ limiting current lines and extrapolation of the bisector to the fitted curvature.

### 3.3 Gallium Determination

The solution of gallium arsenide in nitric acid was evaporated down to virtual dryness, converted to sulfate using 10 ml of water and 1 ml of concentrated ACS reagent grade sulfuric acid, and evaporated down. Ten milliliters of concentrated HCl and 2 ml of H_2_SO_3_ were added and volatilization of AsCl_3_ was carried out on a hot plate at about 80 ° C. H_2_SO_3_ was added two more times and the heating process was continued for a total time of 4 hours. After cooling the solution, NH_4_OH was added dropwise until a precipitate of gallium hydroxide became permanent. An excess of previously standardized Na_2_EDTA · 2H_2_O was weighed into the flask via a powder funnel and washed down with about about 50 ml of pretitrated supporting electrolyte (0.5 M NaCl and 0.5 M sodium acetate; pH = 5.5). The solution was heated for an hour at about 50° C on a hot plate, cooled and delivered into a coulometric cell containing 50 ml of pretitrated supporting electrolyte. Subsequently, the excess EDTA (that amount which did not react with Ga^+++^) was back titrated with electrogenerated Zn^++^, generated coulometrically from the Zn-amalgam anode. The end point was determined amperometrically in the same manner as in the standardization of EDTA. An argon atmosphere was maintained in the cell in the course of the whole experiment.

### 3.4 Arsenic Determination

The sample, pretreated and weighed in the same manner as for the Ga determination, was dissolved in a 125 ml Erlenmeyer flask in 5 ml of concentrated HNO_3_. The excess HNO_3_ was removed by the addition of 1 ml of H_2_SO_4_ and triple evaporation. The pentavalent arsenic was reduced with 5 ml of H_2_SO_3_. The excess SO_2_ was removed by sweeping the flask with nitrogen for one hour. The solution, neutralized with deaerated 1 M NaOH until precipitation of Ga(OH)_3_ became apparent, was delivered via the siphon arrangement into the pretitrated electrolyte (0.1 M KI + saturated solution of NaHCO_3_; pH = 7.0). Titration to the amperometric end point was carried out as described earlier [15]. An argon atmosphere was maintained in the cell.

## 4. Instrumentation^2^

For constant-current coulometric titrations, three different instrumental facilities are required for the measurements of current, time and end-point indicator signal.

### 4.1. Current

A Princeton Applied Research constant-current power supply Model TC602-CR was employed. Its maximum output is 60V and its current rating is 2A. The power supply was operated in a constant-current mode. The current value was selected to be such that the IR drop across the standard resistor equals the voltage of the saturated Weston cell. The standard resistor and Weston cells were calibrated at NBS.

### 4.2. Time

A Beckman Model 3750 Universal EPUT and Timer was used for measuring the time interval between the start and termination of the passage of current through the coulometric cell.

### 4.3. End-Point Determination

A Sargent Model XXI polarograph was used for application of the potential to the indicator electrode system and for recording the indicator current.

### 4.4. Mass Measurement

A Mettler Microbalance was used for weighing. The balance is a constant load type, 20-g capacity. The standard deviation of the mass measurement process on this balance, established over a number of years, is 0.003 mg. The samples were weighed in a platinum boat by the substitution method. NBS calibrated weights were used for this purpose. The weight of the samples was corrected to the mass in vacuum by applying the appropriate corrections for the effect of air buoyancy.

### 4.5. Method of Calibration

The electrical and time measurements were performed against standards calibrated by the appropriate laboratories of the NBS. The accurate current measurement was based on the values of a standard resistor and the voltage of a saturated Weston cell.

## 5. Results

The most direct way of examining the result of stoichiometric determinations is to view them in terms of the number of moles of constituent elements per unit weight of material. Thus, the results of analysis of single crystal GaAs, reported in terms of millimoles of Ga and As per gram of GaAs, are summarized in table 1. The analyses performed show that the analyzed material adheres closely to 1:1 compound stoichiometry. It is important to note here that the determination of stoichiometry through coulometric analysis is totally independent of the knowledge of the atomic weights of the analyzed constituents; for that matter it is independent from most of the constants which are involved in computations (e.g., Faraday’s constant). All known possible systematic errors are believed to be negligible.

Using the stoichiometry data of table 1 and invoking the accepted value for the atomic weight of arsenic (74.9216) the pooled data, reported in table 2, were calculated. The rationale behind the use of pooled data is that no apparent significant difference is found between the molar gallium and arsenic content of the analyzed material. Hence, for 1:1 stoichiometric compound, pooling the molar content values is justifiable and used as the basis for the calculations of the atomic weight of gallium.

Thus, using 74.9216 as the atomic weight of arsenic its concentration in GaAs is found to be 51.7921 percent by weight. Since concentration of impurities in this material was found to be negligibly small, the concentration of gallium can be easily calculated to be 48.2079 percent by weight (as the difference from 100.0000 percent). Hence on the basis of data reported here the atomic weight of gallium is 69.737 ± 0.006, the uncertainty being the 95 percent confidence limit.

## 6. Conclusions

Coulometrically determined concentrations of Ga and As in GaAs confirm a high degree of adherence of the analyzed material to 1:1 compound stoichiometry. The value of the atomic weight of Ga (69.737) computed from data reported here differs somewhat from the IUPAC accepted value of the atomic weight of gallium (69.72) and is believed to be more accurate. It should be noted that this is the first attempt to use GaAs as the material for atomic weight determination. The electrochemical value of the atomic weight of Ga computed here is in a good agreement with the chemical combining weight ratio value obtained by Lundell and Hoffman [4], based on gallium-to-oxygen-weight-ratio in Ga_2_O_3_. Using the two independent values for the atomic weight of Ga (one based on this work and one based on the data of Lundell and Hoffman [4]) the mean chemical value of the atomic weight of Ga based on its compound stoichiometry is 69.735.

It appears that two distinctly different values of the atomic weight of gallium are now in existence. The mass spectrometric value is 69.724 and the chemical value is 69.735. In view of the fact that no significant gallium isotope fractionations are reported in nature, the likelihood of isotope fractionation in the purification of GaAs is highly remote. This apparent discrepancy needs to be resolved. Determination of the atomic weight of gallium coulometrically by another independent approach, similar to that used for the atomic weight of zinc [10, 11], is anticipated in this laboratory. The use of high purity gallium will eliminate a possible source of error in chemical determination of atomic weights, namely that of compound stoichiometry.

## Figures and Tables

**Table 1:** Coulometric Analysis of *GaAs*

Ga, mM/g GaAs	As, mM/g GaAs

6.91291	6.91325
6.91324	6.91283
6.91244	6.91276
6.91269	6.91259
Ave. 6.91282	Ave. 6.91286
S = 0.00034	S = 0.00028
Overall Average = 6.91284	
Pooled standard deviation = 0.00031	
Standard error of overall average = 0.00011	

**Table 2:** Pooled Data^a^

As	51.7921 percent by weight
Ga	48.2079 percent by weight
Atomic weight of Ga = 69.737 ± 0.006	

a These data are based on the pooled stoichiometry data of Table 1 and 74.9216 as the atomic weight of arsenic. The uncertainty indicated is the 95 percent confidence limit.

## Footnotes

1 Figures in brackets indicate the literature references at the end of this paper.

2 Certain commercial equipment, instruments, or materials are identified in this paper in order to adequately specify the experimental procedure. In no case does such identification imply recommendation or endorsement by the National Bureau of Standards, nor does it imply that the material or equipment identified is necessarily the best available for the purpose.
